# Assessing Climate Change Impact on Ecosystems and Infectious Disease: Important Roles for Genomic Sequencing and a One Health Perspective

**DOI:** 10.3390/tropicalmed5020090

**Published:** 2020-06-03

**Authors:** Kenneth B. Yeh, Jeanne M. Fair, Woutrina Smith, Teresa Martinez Torres, Julie Lucas, Corina Monagin, Richard Winegar, Jacqueline Fletcher

**Affiliations:** 1 Global Health Surveillance and Diagnostics, MRIGlobal, Gaithersburg, MD 20878, USA; tmartineztorres@mriglobal.org (T.M.T.); jlucas@mriglobal.org (J.L.); rwinegar@mriglobal.org (R.W.); 2 Biosecurity and Public Health, Los Alamos National Laboratory, Los Alamos, NM 87545, USA; jmfair@lanl.gov; 3 One Health Institute, University of California, Davis, CA 95616, USA; wasmith@ucdavis.edu (W.S.); cmonagin@gmail.com (C.M.); 4 National Institute for Microbial Forensics & Food and Agricultural Biosecurity, Oklahoma State University, Stillwater, OK 74078, USA; jacqueline.fletcher@okstate.edu

**Keywords:** biosecurity, climate change impact, One Health, genome, sequencing, infectious disease

## Abstract

Changes in the Earth’s climate and weather continue to impact the planet’s ecosystems, including the interface of infectious disease agents with their hosts and vectors. Environmental disasters, natural and human-made activities raise risk factors that indirectly facilitate infectious disease outbreaks. Subsequently, changes in habitat, displaced populations, and environmental stresses that affect the survival of species are amplified over time. The recurrence and spread of vector-borne (e.g., mosquito, tick, aphid) human, animal, and plant pathogens to new geographic locations are also influenced by climate change. The distribution and range of humans, agricultural animals and plants, wildlife and native plants, as well as vectors, parasites, and microbes that cause neglected diseases of the tropics as well as other global regions are also impacted. In addition, genomic sequencing can now be applied to detect signatures of infectious pathogens as they move into new regions. Molecular detection assays complement metagenomic sequencing to help us understand the microbial community found within the microbiomes of hosts and vectors, and help us uncover mechanistic relationships between climate variability and pathogen transmission. Our understanding of, and responses to, such complex dynamics and their impacts can be enhanced through effective, multi-sectoral One Health engagement coupled with applications of both traditional and novel technologies. Concerted efforts are needed to further harness and leverage technology that can identify and track these impacts of climate changes in order to mitigate and adapt to their effects.

*Climate change impacts transcend international borders and geographic areas of responsibility*.

CNA Military Advisory Board, National Security and the Accelerating Risks of Climate Change.

## 1. Introduction

**Figure 1:**
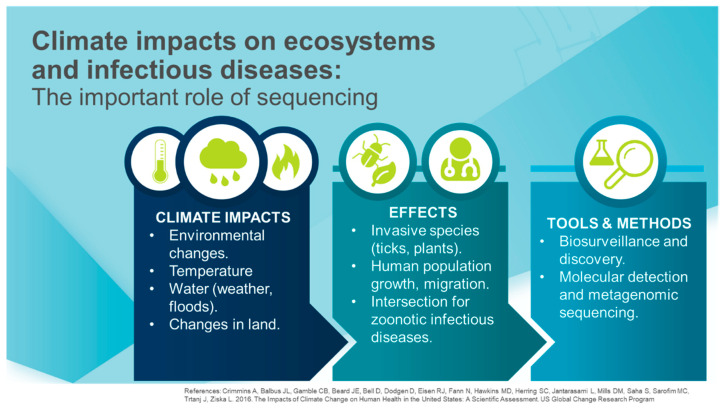
Climate impacts [30] whose effects on ecosystems and infectious diseases can be investigated using a variety of tools.

As part of a panel discussion at the 2019 Sequencing, Finishing, and Analysis for the Future (SFAF) conference (21–23 May, 2019, Santa Fe, New Mexico, USA), we explored the importance of using genomic sequencing in a One Health context to study climate change impacts on natural and managed ecosystems and their infectious diseases (Figure 1). We summarize key discussion topics in which the application of genomic sequencing technologies can enhance our understanding of climate change and infectious diseases using a One Health approach. The topics included an array of scientific applications including current sequencing tools, genomics and environment, science of signatures and conservation genomics, plant ecosystems, a research case study, and outcomes of human behavior such as data and material sharing. The importance of integrating these applications across One Health, a multi-disciplinary, and multi-sectoral approach helps demonstrate how the entire work can be related and interact. Recent effects of relevant infectious diseases linking our discussion to the importance of neglected tropical diseases are also addressed.

Unprecedented opportunities for studying microbial populations have been created by current sequencing technologies [1]. For pathogens with comparatively low per-site mutation rates, such as DNA viruses and bacteria, Biek et al. [1] point out at that whole-genome sequencing can reveal the accumulation of novel genetic variation between population samples taken at different times. The concept of “measurably evolving populations” and related analytical approaches have provided powerful insights for fast-evolving RNA viruses, and sequencing technologies are now economical enough to apply to other DNA microbial pathogens [1]. These methodologies provide unprecedented windows to understanding how pathogens are adapting or evolving in real time in the face of climate change.

Climate change due to higher global temperatures and changing precipitation patterns affects the environment, altering coastlines and exacerbating flooding, and contributes to extreme weather patterns, all of which are influenced by the type, magnitude, and rate of change [2]. Here, climate change impact is defined as those effects resulting from climate change drivers such as deviations from average temperature and precipitation over several decades of time. For example, human, animal and plant systems are affected by rising atmospheric (greenhouse) gases coupled with globalization and other influences including cultural, economic, political, and technological factors [3,4]. Climate change is already affecting populations and infectious disease dynamics among wildlife, plants, insects and other living things, altering host and disease vector life cycles, shifting vector, pathogen and host ranges as well as migratory patterns, along with disease spread and its attributable impacts. The fact that neither transboundary infectious disease agents nor climate impacts respect international borders [5,6] reinforces the need for regional and global approaches that encourage open communication and transparency.

There are multiple scales of impact of changing climatic conditions—the habitats and plants that are responding to a changing climate, the vertebrate organisms that are adapting to the changing habitats and climate, and the microbes that are evolving to the new conditions at each level. The core thread between each of these biological scales is the genome. Genomes in all organisms evolve and adapt to new conditions over time. The genome may change by gene expressions in response to the environment or there may be modifications in the genetic code itself. Following the changes in the genomes of organisms at the multiple scales in response to changing conditions requires the measurement of the genome, which is primarily accomplished through next generation sequencing. In the One Health construct of considering the health of humans, animals, and the environment together, the genome is a foundational element for measuring the adaptation these systems over time.

Environmental changes caused by natural disasters and human-made accidents raise risk factors that indirectly increase infectious human disease outbreaks as a result of displaced human populations, constrained resources, and damaged infrastructure [7]. When variables including health, socio-economic, and political factors are considered together, climate change and its impacts can be thought of as “threat multipliers” [8,9]. Addressing these challenges through a holistic One Health approach recognizes and integrates perspectives related to animals, humans, and environment [8]. Key to the environmental aspect of the One Health concept is the importance of plant systems, which provide food and fiber and support the economy, contributing to socio-economic and political stability [3,10]. Both agricultural and environmental change are inextricably linked to climate change and, like higher vertebrate species, microbial pathogens will either adapt or not.

The design of effective programs for scientific inquiry, modeling, response to and mitigation of climate change impacts on biological systems will require a multi-sectoral, cross-disciplinary approach. For example, plant systems are often omitted from One Health considerations despite their direct nutritional and ecological relationships to animals and humans. Here, we note that plants and their phytobiomes have critical roles in both food security and climate change. One consequence is that the loss of agricultural productivity resulting from climate change can impact food security directly (reduced food production) or indirectly (the impact of rising global temperatures on food access, utilization, and safety) [11]. Furthermore, there is a link between food security, social stability and national security [3], as inadequate food supplies may trigger social unrest and/or human movement and migration. The impacts of these changes may be most severe among populations that are less affluent and those in the tropics [3,11,12]. Addressing these issues must involve key players from the climate, environmental, and genomics sciences and members of the security sector. In addition, public-private partnerships will help to galvanize national government level activity with those of industry and civil society.

## 2. Current State of Sequencing Tools

Tools and associated methods available for One Health-based responses to climate change impacts include both gold standard quantitative real-time PCR (qPCR) and current metagenomics sequencing approaches, which are less biased [13]. Their application can provide increased ability to detect and identify emerging and new pathogens and biological invasion by invasive species that are expected to increase due to climate impact [14,15]. Recently, the newly emerged coronavirus, which was first called the 2019-novel coronavirus (2019-nCoV) and now designated as the severe acute respiratory syndrome coronavirus-2 (SARS-CoV-2), was first reported in four cases associated with a wholesale market in Wuhan, China on 29 December 2019; [16]. The speed and transparency of the early work to understand this newly emerged coronavirus is remarkable; the full genome was released to the world on 10 January 2020. The release of the SARS-CoV-2 genome led to an almost real-time understanding of the viral phylogenetics, and the deduction that it came from a wildlife wet market, jumping from animal species [17] commonly bought at the market for consumption. With human populations, wildlife reservoirs, vectors, and microbes adapting in response to rapidly changing environmental conditions, outbreaks of pandemic potential such as that caused by SARS-CoV-2, will occur more regularly.

Genomic sequencing can be applied across many disciplines which are also relevant to climate change impacts, One Health, and biosecurity. A chart detailing a framework of how a variety of genomic tools can be applied to better understand various aspects of biological invasions [15,17] can help to facilitate outbreak response, enhance understanding of the role of changing environment and facilitate management of priorities through each stage of the invasion process. This framework of tools is also relevant to tracking certain neglected tropical diseases such as schistosomiasis, which is caused by parasitic flatworms and transmitted by exposure to contaminated water sources. Freshwater snails serve as the intermediate host of the flatworms. Climate change is expected to continue drier and hotter conditions across many parts of Africa, which should decrease the incidence of schistosomiasis by reducing the extent and availability of snail habitats [18]. Genomic sequencing can be applied here to track and multiple organisms and detect the presence of various species within a given habitat.

Developing high-quality pathogen detection methods used in clinical diagnostics, infectious disease surveillance, and forensics requires an understanding of the true genetic diversity of the pathogens, their ecology, geographic distribution, reservoirs, hosts, vectors, and genetically related organisms. Using a priori approaches to design molecular detection assays can lead to signature erosion. In the H1N1 influenza pandemic of 2009, for example, a genetic shift in the pathogen resulted in the inability of existing PCR assays to detect that strain [19]. The more we look at the genetic diversity of a particular pathogen, the more we discover, including unique sequences that reduce the sensitivity or specificity of existing diagnostics or detection methods.

Pathogen genetic diversity can be a hindrance to assay development as in the case of Lassa fever, which has symptoms similar to malaria and viral hemorrhagic fevers such as Ebola, and related co-infections among patients. Due to the complex diversity of the Old World arenavirus causing Lassa fever, PCR assays specifc for detection of this virus are difficult to design despite their single transmission mode via contact with rodent reservoirs [20]. This is due to the large genetic diversity of viral sequences in the reservoir population. Each cluster of human disease outbreak appears to derive from a single rodent-to-human transmission event that randomly samples a small portion of the true viral genetic diversity. Therefore qPCR assays are often designed using a snapshot of the viral genetic diversity known available at the time. Subsequent disease outbreaks may reveal new genetic diversity, eroding the performance of existing assays. A comprehensive surveillance of the rodent reservoir would be needed to understand the true level of genetic diversity that assays should be designed to detect. The need for proper biosafety controls from secure sample transport to laboratory biocontainment especially in low resource areas reinforce the need for effective, timely, and low-cost diagnostics [21].

## 3. Genomics and a Changing Environment

Although both climate change impacts and the research needed to understand and mitigate them are long term, time is no longer on our side and our approaches to solving such complex problems will require creativity and expediency. In epidemiological or climatological modeling, for example, data collection is traditionally completed first, followed by model development. However, a recent trend is for the two to be implemented concurrently so that data gaps are recognized immediately and data collection can target those gaps in real time. Estimating population spread and impacts of scenarios for changing environments or newly emerging viruses is now possible given advances in ecologic and epidemiologic models [22,23]. Genetic research and sequencing analyses have shown correlations suggesting that invasive species (weeds) moving into agricultural landscapes require less flexibility and less genetic diversity than those moving within the natural environment [15]. Tracking plant movements can help to inform the development of tools for predicting future plant invasions and migrations, which are expected to increase as climate change-induced habitat disturbances become more widespread [15]. Such approaches increase our ability to predict shifts and trends, whether in climate changes or pathogen spillover.

With regard to sequencing technology and meeting the challenge of climate change, there are two primary ways that genomics can be applied immediately. The first, as mentioned above, is understanding host, vector and pathogen range shifts due to environmental change. The second is incorporating sequencing of archived sample sets to investigate how pathogens are both evolving and shifting into new hosts and ranges. Recent evidence finds climate change is already affecting wildlife populations and disease dynamics [24,25,26,27], altering host and disease vector life cycles as in case of certain NTDs, shifting vector and host ranges and migratory patterns [26,28], and changing disease spread and its impacts. The consequences of these climatically induced changes are poorly understood, generating the need for comprehensive research involving both genomics [27] and predictive modeling of infectious disease and species distributions [29]. A multitude of factors may influence how both hosts and vectors of infectious diseases respond to changing climates. Finding signatures of environmental change in biological communities, as well as fusing and analyzing multi-scale data across species, time, and latitude, will be key.

Combining sequencing and modeling will allow us to better understand how climate may be affecting geographic ranges of hosts, human migration, vectors, and parasites. The following questions can be asked using newly collected or archived samples from freezers around the world: How have pathogens in vectors and hosts changed over the last several decades? How do pathogens differ, at the genomic scale as well as in prevalence, geographically in both the hosts and vectors? Answering these sorts of questions is possible by simultaneously using sequencing with epidemiological models to make predictions that incorporate characteristics of pathogens, populations, and environmental variables.

## 4. Science of Signatures and Conservation Genomics

In order to understand the natural world, we must first find patterns that are otherwise invisible or indistinguishable from the chaos of natural systems. Finding such patterns can reveal signatures of long-term or short-term impacts of environmental events, evolutionary processes, or other idicators of environmental health. Using genomic sequences and bioinformatics to find and measure signatures in organisms can contribute to enhanced understanding of these complex systems and to better prediction of future impacts. In understanding the impacts of climate change on species, scientists are working to understand and predict which species will be able to adapt locally and which species will not be able to adapt [31,32]. Many of these adaptations will be through genetic adaptation to a changing environment [33].

Both marine and terrestrial ecosystems are under pressure, not only from climate change, but from over-exploitation, habitat loss and degradation, growing human populations, and ultimately species imperilment. Global climate change is altering our ecosystems at an unprecedented rate, threatening plants, animals, and the habitats in which they live. According to five coordinated reports in 2019 from the Intergovernmental Science-Policy Platform on Biodiversity and Ecosystem Services (IPBES), sponsored by the United Nations, over 1 million species are threatened with extinction [34]. Conservation genomics, which is closely linked to understanding the health of individuals and populations, can inform our efforts to manage infectious diseases and animal and plant health under these new environmental challenges. Understanding how ecosystems, and the populations within them, are likely to respond under different climate scenarios is essential for planning future conservation strategies and mitigations for infectious diseases. Modernizing our approach to natural resource management will ensure that we are utilizing the best available science to help make complex and difficult management decisions, and embracing sequencing where appropriate to find those signatures of the health of populations will be as important as the application of sequencing towards infectious disease diagnosis and detection.

## 5. Plant Ecosystems

In both natural and agro-ecosystems, plants play critical ecological roles by utilizing carbon dioxide and releasing oxygen, emitting substrates into the soil and atmosphere, modifying soil moisture, and reducing the amount of rainfall reaching the ground [35]. Effective assessment of climate issues related to plants should consider the plant as a complex “phytobiome” composed of communities of species within an environmental niche [36], and aspects of climate change, particularly altered temperatures and rainfall, modify community diversity [3]. Crop plants are cultivated in disturbed ecosystems and, at least in developed nations, are typically planted in monocultures, adding to their vulnerability to attack by new species of invasive pests or pathogens.

Since infectious disease develops only in a conducive environment [37], climate change will be a key driver of changes in plant disease epidemiology. For example, even a small increase in the length of a growing season can significantly increase the amount of pathogen inoculum produced [38]. Some genes encoding host plant disease resistance factors are less effective at higher temperatures, and higher cool-season temperatures may favor the survival of insect vectors of some plant pathogens [38]. Changes in climate (e.g., higher temperatures, lack of water, increased carbon dioxide, and other environmental changes) impact each of these elements in different ways including environmental hardiness, changes in developmental rates and flowering times, productivity, shifts in geographic ranges, and virulence (for pathogens and other microbes) or disease susceptibility (for plants) [15,35].

## 6. Research Example and Case Study of One Health Approaches to Using Sequencing to Understand and Mitigate Climate Change Impacts

Sequencing can provide a unique opportunity to derive new information from archived sample sets, especially when historical and recent samples are compared directly. Investigation can focus on genomic changes in individual microbes or microbiomes, or from host organisms. Targeted approaches can select specific pathogens and identify how they are associated with environmental change.

*Vibrio cholerae* is a well-known model for studying water-borne diseases and as part of an ecosystem, combined with environmental dynamics has been termed as biocomplexity [39]. When combined with genomic sequencing, epidemiology, and remote sensing, non-cholera vibrios such as *Vibrio parahaemolyticus* and *Vibrio vulnificus* represent a microbial barometer for climate change [40]. In 2016–2017, genomic sequencing showed that isolates in Yemen were *V. cholerae* serotype Ogawa isolates from a single sublineage of O1 El Tor [41]. This outbreak in Yemen which resulted in over one million cases and over 2000 deaths exemplifies the intersection of climate change, human disease, and related social factors of conflict and migration.

Zoonotic dieases such as plague and hantavirus caused by *Yersinia pestis* bacterium and Sin Nombre virus, respectively, are endemic in the US Four Corners region (Utah, Colorado, Arizona, and New Mexico). While the transmission of plague and hantavirus are different, the incidence of these viruses depends on rodent reservoirs and vectors (e.g., fleas). The occurrence of heavy precipitation in a given year often results in a larger rodent population, which increases risk of hantavirus transmission through interaction with humans. Models for predicting zoonotic diseases include diverse reservoirs of rodents in Central Asia, the Middle East, and the US Midwest which is adjacent to the Four Corners region [42]. Overall, the ability to collect and reconcile historical data with predictive modeling will be very powerful to understand transmission dynamics.


**Case-Study: Expanding New Sequencing Technology Across the Globe to Detect Animal to Human Disease Spillover**
  Climate and health are intertwined with the effects of climate shifts that are currently manifesting globally. New technology, specifically in sequencing and metagenomics, must be implemented in geographically diverse regions around the globe in order to maximize impact. These newly developed or newly implemented technologies have the capacity to benefit even remote populations, who are often the most impacted by these health shifts. In addition to the identification of new pathogens, training on use of these technologies will have long-term impact by building local capacity to detect and respond to emerging health threats on a routine basis.  The USAID-funded PREDICT program [43,44] was implemented in over 30 countries around the globe from 2009–2020, aimed to strengthen capacity for surveillance and detection of viral threats transmitted from animals to humans. In order to address challenges of building and sustaining laboratory viral detection capacity in low-resource partner countries, the PREDICT approach built laboratory platforms based on conventional PCR methods to identify potential pathogens in wildlife and humans. Building upon this approach, the project also integrated more complex sequencing technology to empower local partner universities and governments to increase their viral detection capacity at the local level. Partner countries who successfully adopted this approach identified over 1000 new viruses at the human-animal interface. Recognition of the significant scientific and economic benefits of discovering high-consequence pathogens before evidence of spill-over into human populations, participating countries are now starting to realize the power and importance of sequencing and prioritize this technology as a key component to future capacity building [45]. This new focus in low-resource, viral hot-spot regions of the world promotes the potential for additional high-level metagenomic skills to be added to the cadre of possibilities. A critical piece of continuing the work of PREDICT is to encourage countries to store sample repositories for the long-term so that further work can be done utilizing mores powerful metagenomic approaches as they become available. As these countries are particularly impacted by drivers such as climate shifts, human and animal movement, and population growth, we anticipate continuing viral spillover at the human-animal interface, making the need for a continued increase and investment in pathogen detection capacity [46].  The sequencing technologies discussed in this paper, which expand our ability to detect pathogens of pandemic potential, should be deployed locally and globally in areas where spillover and disease spread continue to occur. The challenge is to continue a global dialogue to encourage development of affordable platforms and training that can lead to discovery of known and novel viruses with pandemic potential [47]. The One Health Institute at the University of California, Davis, led the PREDICT Project and is also home of the University of California Global Health Institute’s Planetary Health Center of Expertise that works to raise awareness about climate and health issues as well as sustainable solutions and platforms (https://ohi.vetmed.ucdavis.edu). With the help of such centers, the global population at large is beginning to appreciate the importance of understanding local predictions for environmental change and disease spillover to utilize adaptation and mitigation measures. It has become well known that understanding drivers of pathogen spillover and risk, and utilizing state-of-the art technology for management of these threats, are critical. The authors are optimistic that there is a true space for development of easily accessible technologies that are farm, clinic or animal/human-specific, resulting in rapid, real-time on-site diagnostic care utilizing the new sequencing technologies discussed herein.

## 7. Importance of Trust and Sharing of Data and Material

There is a continued need to earn and build trust between partners at the working level of research groups in universities, companies and institutes, and the top level of government partners especially in host countries where work is being implemented. It is critical to invest time to build a collaborative network that respectfully supports trust through transparency and partnership. The ability to collaborate professionally with various partners is critical to generate and provide the various forms of data and technology (from field to lab), including the models and sequencing methods discussed herein. More frequently, funding and donor agencies are requiring data be made available in a public and open source manner. Occasionally frameworks for data sharing are provided, but other times the research team needs to develop an approach that works best to meet the requirements and needs. The ability to work in a global society beyond any individual research lab, or any individual organization to generate a solution by working together should be recognized. One key to success is to have strong partnerships on the ground with protocols for data sharing in place from the beginning.

## 8. Conclusions

Through our discussion, we draw attention to the power of genomic sequencing that complements expertise and tools across disciplines and sectors to address climate change impacts on ecosystems and infectious diseases. Using a One Health approach is important to view the problem on a larger scale, and beyond the scientific and technical work the government policies developed in some countries are now starting to take a coordinated One Health multi-sectoral approach to implement change. We described some examples of climate change impacts on animal and human infectious disease that exist in vector-borne, zoonotic, and neglected tropical diseases. Climate change is also a key driver of multiple changes in plant epidemiology, affecting disease susceptibility and resistance as well as environmental hardiness, developmental and flowering rates, productivity, and geographical range. Genetic tools facilitate the prediction of such changes and help in the development of management options. Shaping human behavior is key to positive and practical outcomes. Besides the importance of shared goals and trust-building as related to setting research agendas and sharing intellectual resources, another critical theme that arose is the need to increase partnering, especially for engaging public and private partners that can help incorporate a multi-sectoral approach with a diverse audience of stakeholders who will form policy. This audience goes beyond public and animal health specialists and targets traditional biotechnology as well as social and life scientists that have expertise in behavior change, food safety and security, the environment, hydrology, climate, and meterology. The practice and policies developed will shape funding which, in turn, will leverage the commercial partnerships that are challenged with pursuing markets with varying returns on investments and are also critical in sustaining programs through the economy and enterprise. This combination of resources and expertise contributes to a more open and collaborative approach to understanding the links between infectious disease and climate.

## References

[1] Biek R., Pybus O.G., Lloyd-Smith J.O., Didelot X. (2015). Measurably Evolving Pathogens in the Genomic Era. Trends Ecol. Evol..

[2] Marjoram T. (2016). Silk Roads for the 21st Century: Engineering Mega-Infrastructure for Development and Sustainability. Front. Eng. Manag..

[3] Stack J.P., Fletcher J., Gullino M.L., Mass A., Bodó B., Burnley C., Comardicea I., Roffey R. (2013). Climate Change and Plant Biosecurity: A New World Disorder?. Global Environmental Changes: New Drivers for Resistance, Crime and Terrorism.

[4] Mirski T., Bartoszcze M., Bielawska-Drózd A. (2012). Impact of Climate Change on Infectious Diseases. Pol. J. Environ. Stud..

[5] CNA Military Advisory Board National Security and the Accelerating Risks of Climate Change. https://www.cna.org/cna_files/pdf/MAB_5-8-14.pdf.

[6] Siembieda J.L., Kock R.A., McCracken T.A., Newman S.H. (2011). The Role of Wildlife in Transboundary Animal Diseases. Anim. Health Res. Rev..

[7] Kouadio I.K., Aljunid S., Kamigaki T., Hammad K., Oshitani H. (2012). Infectious Diseases Following Natural Disasters: Prevention and Control Measures. Expert Rev. Anti. Infect. Ther..

[8] Essack S.Y. (2018). Environment: The Neglected Component of the One Health Triad. Lancet Planet. Health.

[9] Black P.F., Butler C.D. (2014). One Health in a World with Climate Change. Rev. Sci. Tech..

[10] Fletcher J., Franz D., Leclerc J.E. (2009). Healthy Plants: Necessary for a Balanced “One Health” Concept. Vet. Ital..

[11] Brown M.E., Antle J.M., Backlund P., Carr E.R., Easterling W.E., Walsh M.K., Ammann C., Attavanich W., Barrett C.B., Bellemare M.F. (2015). Climate Change, Global Food Security and the U.S. Food System.

[12] Ghini R., Bettiol W., Hamada E. (2011). Diseases in Tropical and Plantation Crops as Affected by Climate Changes: Current Knowledge and Perspectives. Plant Pathol..

[13] Yeh K.B., Wood H., Scullion M., Russell J.A., Parker K., Gnade B.T., Jones A.R., Whittier C., Mereish K. (2019). Molecular Detection of Biological Agents in the Field: Then and Now. mSphere.

[14] Mansfield K.L., Jizhou L., Phipps L.P., Johnson N. (2017). Emerging Tick-Borne Viruses in the Twenty-First Century. Front. Cell. Infect. Microbiol..

[15] Chown S.L., Hodgins K.A., Griffin P.C., Oakeshott J.G., Byrne M., Hoffmann A. (2015). Biological invasions, climate change and genomics. Evol. Appl..

[16] Qun L. (2020). An outbreak o f NCIP (2019-nCoV) infection in China—Wuhan, Hubei province, 2019–2020. China CDC Wkly..

[17] Ji W., Wang W., Zhao X., Zai J., Li X. (2020). Cross-species Transmission of the Newly Identified Coronavirus 2019-nCoV. J. Med. Virol..

[18] Blum J.A., Hotez P.J. (2018). Global ‘worming’: Climate change and its projected general impact on human helminth infections. PLoS Negl. Trop. Dis..

[19] Klungthong C., Chinnawirotpisan P., Hussem K., Phonpakobsin T., Manasatienkij W., Ajariyakhajorn C., Rungrojcharoenkit K., Gibbons R.V., Jarman R.G. (2010). The impact of primer and probe-template mismatches on the sensitivity of pandemic influenza A/H1N1/2009 virus detection by real-time RT-PCR. J. Clin. Virol..

[20] Mazzola L.T., Kelly-Cirino C. (2019). Diagnostics for Lassa fever virus: A genetically diverse pathogen found in low-resource settings. BMJ Glob. Heal..

[21] Taboy C.H., Chapman W., Albetkova A., Kennedy S., Rayfield M.A. (2010). Integrated Disease Investigations and Surveillance Planning: A Systems Approach to Strengthening National Surveillance and Detection of Events of Public Health Importance in Support of the International Health Regulations. BMC Public Health.

[22] Plowright R.K., Eby P., Hudson P.J., Smith I.L., Westcott D., Bryden W.L., Middleton D., Reid P.A., McFarlane R.A., Martin G. (2014). Ecological dynamics of emerging bat virus spillover. Proc. R. Soc. B..

[23] Riou J., Althaus C.L. (2020). Pattern of early human-to-human transmission of Wuhan 2019 novel coronavirus (2019-nCoV), December 2019 to January 2020. Eurosurveillance.

[24] Matthysen E., Adriaensen F., Dhondt A.A. (2011). Multiple Responses to Increasing Spring Temperatures in the Breeding Cycle of Blue and Great Tits (Cyanistes Caeruleus, Parus Major). Glob. Chang. Biol..

[25] Melles S.J., Fortin M.J., Lindsay K., Badzinski D. (2011). Expanding Northward: Influence of Climate Change, Forest Connectivity, and Population Processes on a Threatened Species’ Range Shift. Glob. Chang. Biol..

[26] Carey C. (2009). The Impacts of Climate Change on the Annual Cycles of Birds. Philos. Trans. R. Soc. B Biol. Sci..

[27] Ruegg K., Bay R.A., Anderson E.C., Saracco J.F., Harrigan R.J., Whitfield M., Paxton E.H., Smith T.B. (2018). Ecological Genomics Predicts Climate Vulnerability in an Endangered Southwestern Songbird. Ecol. Lett..

[28] Bridge E.S., Kelly J.F., Bjornen P.E., Curry C.M., Crawford P.H.C., Paritte J.M. (2010). Effects of Nutritional Condition on Spring Migration: Do Migrants Use Resource Availability to Keep Pace with a Changing World?. J. Exp. Biol..

[29] Bartlow A.W., Manore C., Xu C., Kaufeld K.A., del Valle S., Ziemann A., Fairchild G., Fair J.M. (2019). Forecasting Zoonotic Infectious Disease Response to Climate Change: Mosquito Vectors and a Changing Environment. Vet. Sci..

[30] Crimmins A., Balbus J.L., Gamble C.B., Beard J.E., Bell D., Dodgen D., Eisen R.J., Fann N., Hawkins M.D., Herring S.C., USGCRP (2016). The Impacts of Climate Change on Human Health in the United States: A Scientific Assessment.

[31] Atkins K.E., Travis J.M.J. (2010). Local Adaptation and the Evolution of Species’ Ranges under Climate Change. J. Theor. Biol..

[32] Macfadyen S., McDonald G., Hill M.P. (2018). From Species Distributions to Climate Change Adaptation: Knowledge Gaps in Managing Invertebrate Pests in Broad-Acre Grain Crops. Agric. Ecosyst. Environ..

[33] Somero G.N. (2010). The Physiology of Climate Change: How Potentials for Acclimatization and Genetic Adaptation Will Determine “Winners” and “Losers”. J. Exp. Biol..

[34] Intergovermental Science-Policy Platform on Biodiversity and Ecosystem Services IPBES Global Assessment. https://ipbes.net/news/ipbes-global-assessment-preview.

[35] Dutta H., Dutta A. (2016). The Microbial Aspect of Climate Change. Energ. Ecol. Environ..

[36] Phytobiomes Writing Team Phytobiomes: A Roadmap for Research and Translation. http://www.phytobiomes.org/roadmap/Pages/default.aspx.

[37] Agrios G. (2005). Plant Pathology.

[38] Garrett K.A., Forbes G.A., Savary S., Skelsey P., Sparks A.H., Valdivia C., van Bruggen A.H.C., Willocquet L., Djurle A., Duveiller E. (2011). Complexity in Climate-Change Impacts: An Analytical Framework for Effects Mediated by Plant Disease. Plant Pathol..

[39] Constantin de Magny G., Colwell R.R. (2009). Cholera and climate: A demonstrated relationship. Trans. Am. Clin. Climatol. Assoc..

[40] Baker-Austin C., Trinanes J., Gonzalez-Escalona N., Martinez-Urtaza J. (2017). Non-Cholera Vibrios: The Microbial Barometer of Climate Change. Trends Microbiol..

[41] Weill F.X., Domman D., Njamkepo E., Almesbahi A.A., Naji M., Nasher S.S., Rakesh A., Assiri A.M., Sharma N.C., Kariuki S. (2019). Genomic insights into the 2016–2017 cholera epidemic in Yemen. Nature.

[42] Han B.A., Schmidt J.P., Bowden S.E., Drake J.M. (2015). Rodent reservoirs of future zoonotic diseases. Proc. Natl. Acad. Sci. USA.

[43] Kelly T.R., Karesh W.B., Johnson C.K., Gilardi K.V.K., Anthony S.J., Goldstein T., Olson S.H., Machalaba C., Mazet J.A., Predict Consortium (2017). One Health proof of concept: Bringing a transdisciplinary approach to surveillance for zoonotic viruses at the human-wild animal interface. Prev. Vet. Med..

[44] Bird B.H., Mazet J.A.K. (2018). Detection of Emerging Zoonotic Pathogens: An Integrated One Health Approach. Annu. Rev. Anim. Biosci..

[45] Montecino-Latorre D., Goldstein T., Gilardi K., Wolking D., Van Wormer E., Kazwala R., Ssebide B., Nziza J., Sijali Z., Cranfield M. (2020). Reproduction of East-African bats may guide risk mitigation for coronavirus spillover. One Health Outlook.

[46] Carroll D., Daszak P., Wolfe N.D., Gao G.F., Morel C.M., Morzaria S., Pablos-Méndez A., Tomori O., Mazet J.A.K. (2018). The Global Virome Project. Science.

[47] Goldstein T., Anthony S.J., Gbakima A., Bird B.H., Bangura J., Tremeau-Bravard A., Belaganahalli M.N., Wells H.L., Dhanota J.K., Liang E. (2018). The discovery of Bombali virus adds further support for bats as hosts of ebolaviruses. Nat. Microbiol..

